# 

**Published:** 1988-08

**Authors:** 


**Editor**
Mr M. G. Wilson
*40 Coombe Lane, Bristol BS9 2BJ*
*Tel: 0272-682320*

**Assistant Editor**
Dr P. R. Goddard

**Editorial Committee**
Mr B. P. Jones (Secretary),
*Medical Librarian, Bristol University*
Dr J. L. Burton

**Correspondents**
Dr A. E. Adam (Taunton)
Mr D. C. C. Bartolo
Mr M. L. Crosfill (Penzance)
Dr J. D. Davies
Dr J. Jancar
Professor D. J. Pereira Gray (Exeter)
Dr H. G. Mather
Professor G. M. Stirratt
Mr C. Teasdale (Plymouth)
Dr J. L. G. Thomson
Dr M. J. Whitfield
Professor G. K. Wilcock

**BRISTOL MEDICO-CHIRURGICAL SOCIETY**

**President**
Dr H. M. Norman

**Secretary**
Mr R. N. Baird,
*23 Old Sneed Park, Bristol BS9 1RG*

**Treasurer**
Dr N. C. Tricks,
*The Mill, Wrington, Bristol*

**Published by**
**Clinical Press,**
*Greinton House,*
*8 Exeter Buildings,*
*Redland,*
*Bristol BS6 6TH*

**Typesetting by**
*Avon Communications Ltd.,*
*Avon House, Nailsea, Bristol BS19 2DJ*

**Printed by**
*Suttons, Paignton*

Established 1883

### BRISTOL MEDICO-CHIRURGICAL JOURNAL

**AUGUST 1988**
**Volume 103 (iii)**

Copyright © Clinical Press Ltd 1988.

Published quarterly and distributed free to all doctors in the West of England. Overseas subscription £12.

What I know remains unknown, unless by me to other shown.
*Lucilius (180–102 BC)*

### BRISTOL MEDICINE

**NOTICE TO CONTRIBUTORS**
Contributions are invited especially from West of England authors on a variety of subjects, e.g. Original articles relating to the practice of medicine, reports on the contemporary medical scene, obituaries, historical accounts, recollections of colleagues and events worthy to be remembered and liable to be forgotten. An article of 2,000 words with 2 illustrations will occupy 2 pages and this is a suggested, though by no means an obligatory length. Articles are preferred in the form proposed by the International Committee of Medical Editors (Vancouver System)—pamphlet obtainable from Editor of BMJ., BMA. House, Tavistock Square, London, WC1H 9JR.

